# Sensory Reactivity in Autism: Integrating Behavioural, Affective, Physiological, and Neural Dimensions

**DOI:** 10.1007/s11920-026-01682-4

**Published:** 2026-06-09

**Authors:** Teresa Tavassoli, Elysa J. Marco, Nick Puts

**Affiliations:** 1 University of Reading, Harry Pitt Building, Earley Gate, Reading, RG6 6ES UK; 2 Cortica Healthcare, 4000 Civic Center Dr Ste 100, San Rafael, CA 94903 USA; 3 Department of Forensic and Neurodevelopmental Sciences, Institute of Psychiatry, Psychology, and Neuroscience, King’s College London, 16 De Crespigny Park, London, SE5 8AB UK

**Keywords:** Sensory reactivity, Autism, Behavioural, Affective, Physiology

## Abstract

**Purpose of Review:**

The goal of this paper is to synthesise recent research on sensory reactivity differences in autism across the lifespan, using He et al.’s sensory taxonomy as an organising framework. The review aims to address how behavioural, affective, perceptual, physiological, and neural levels of processing contribute to sensory reactivity differences, and how these differences relate to broader outcomes such as mental health, adaptive functioning, and quality of life.

**Recent Findings:**

Across behavioural studies, autistic youth show elevated and variable sensory responsivity, with hypersensitivity predicting internalising symptoms and sensory seeking linked to externalising behaviours. Affective reactivity is consistently elevated across cultures, associated with anxiety and caregiver stress, and sensory seeking may function as a coping mechanism.

Psychophysical research reveals domain‑specific perceptual differences—such as reduced tactile adaptation, altered motion noise exclusion, and enhanced pitch discrimination—rather than overarching hyper‑ or hyposensitivity. These perceptual findings often show limited correspondence with questionnaire‑based measures. Physiologically, autonomic dysregulation is implicated, or pharmacological approaches show emerging promise. Neuroimaging evidence highlights excitation–inhibition imbalance and altered connectivity, including dissociations between exogenous and endogenous networks in sensory‑reactive autistic children.

**Summary:**

Across multiple levels of processing, sensory reactivity differences in autism are robust, heterogeneous, and meaningfully linked to mental health and daily functioning. Key conclusions include:

• Sensory hyperreactivity predicts internalising challenges, while sensory seeking may reflect regulatory strategies.

• Perceptual differences are domain‑specific

• Physiological and neural evidence converges on autonomic dysregulation and differences in connectivity patterns.

## Introduction

Sensory reactivity differences are common in autistic individuals across the lifespan from children [1, 2] to adults [3]. In fact, over 94% of autistic adults report sensory hyperreactivity (e.g. a strong response to sensory stimuli such as being overwhelmed by sounds), while 40% reporting sensory seeking (e.g. a craving for sensory stimuli, such as being fascinated by spinning objects) and 30% report hyporeactivity (e.g. a slow or no response to sensory stimuli where one might be expected) [4]. Importantly, Bradshaw et al., [3] found that autistic adults self-report significantly more sensory needs than informants with intellectual disabilities, suggesting that behavioural responsivity may be under-recognised in adult populations with limited communication [3]. In paediatric studies, it is clear that sensory reactivity does not only occur in autism. Overall 50% of children diagnosed with attention disorders also experience sensory hyperreactivity and 30% of children with additional needs have sensory reactivity differences. With an estimated 5–15% of kindergarteners experiencing sensory reactivity differences, it is also clear that how information from the outside world is being processed in the brains is critical to understand for all humans at any age and with associated neurologic or mental health conditions. While the field has moved forward tremendously over the past 50 years in terms of understanding the aetiologies and consequences of sensory reactivity differences, there is still much to be learned in order to improve the lives of affected individuals including understanding how to modify environments, including homes, schools, and communities, to be sensory supportive. Here we provide an overview of research conducted over the last 5 years in the field of sensory processing in autism. We will follow He et al., [5] sensory taxonomy with 5 levels of sensory processing, starting at behavioural responsivity (J. L. He et al., [5].

## Behavioural Responsivity

Behavioural responsivity, as defined by He et al.,’s sensory taxonomy, refers to observable reactions to sensory stimuli, distinct from perceptual sensitivity and physiological reactivity (J. L. He et al., [5]. This dimension is particularly relevant in autism, where individuals often exhibit different behavioural responses across sensory contexts. Using the Sensory Processing 3-dimensional Observation assessment (SP3-D), Chaturverdi et al., (2025) demonstrated that autistic youth show elevated and more variable sensory responsivity compared to typically developing peers, with observed sensory hyperreactivity persisting through adolescence [6]. Notably, parent-reported and observed measures were not correlated, underscoring the importance of multi-method approaches in assessing behavioural responsivity.

Cross-cultural findings further highlight the variability of behavioural responsivity. Alsaedi et al., [1] reported elevated sensory processing difficulties across modalities in children with autism in the Gulf region [1]. Worthley et al., [7] showed that early sensory responsivity profiles predicted later adaptive functioning, particularly sensory hypersensitivity predicted lower socialisation, reinforcing the developmental significance of behavioural responsivity in autism [7]. Similarly, Rossow et al., [8] used a sensory observational measure that captures behavioural responsivity, the SAND (Sensory Assessment for Neurodevelopmental Differences). They found that in autistic children hyperreactivity was associated to internalising problems such as anxiety and depression, and sensory seeking was linked to externalizing symptoms such as hyperreactivity. Surprisingly few studies have examined sensory responsivity in adults at such a level of granularity.

### Affective Reactivity

Affective reactivity, as defined in He et al.,’s sensory taxonomy, refers to the emotional responses elicited by sensory stimuli, which may be different in autistic individuals (J. L. He et al., [5]. This domain is critical for understanding how sensory experiences intersect with emotional regulation and social functioning. Studies using sensory questionnaires such as the Sensory Profile, which is mainly measuring affective reactivity, e.g. reacting emotionally to touch, show differences in autistic youth [9].

Building on the fundamental first-person accounts, recent qualitative studies provide insights into the lived affective dimensions of sensory experiences. MacLennan et al., [4] highlights the complexity of sensory processing in autistic adults, with participants describing sensory environments as overwhelming, distressing, or even painful—experiences often invisible to others [4]. Emerging data shows that the mental health consequences of sensory experiences are not only significant for the affected individual but for caregivers as well [10]. Talcer et al., [11] extends this to autistic mothers, who reported sensory overload as a source of emotional exhaustion and social withdrawal [11].

It is important to consider the impact that sensory reactivity differences can have. Several studies have reported impacts on mental and physical health [4, 8, 12]. Recent work [13] also reported links to anxiety, with autistic adults perceiving sensory hyperreactivity to precede anxiety and sensory seeking being more an effect of anxiety. This also highlights that sensory seeking might be used as a coping strategy, distinct from reactivity. Sensory seeking behaviours have however also been linked to cognitive outcomes. In autistic males, higher sensory seeking, as measured using the Sensory Profile, was associated with poorer sustained attention performance, indicating that sensory profiles may influence attentional regulation [14]. Kadlaskar et al., [15] reported that autistic young children with autism who showed elevated sensory hyperreactivity also demonstrated lower adaptive functioning and increased attentional problems, indicating that affective responses to sensory input may influence other areas of development.

Affective reactivity is seen across cultures in autism. Reda and colleagues [16], studying an Egyptian sample using the Short Sensory profile, found that affective reactivity differences were common in Egyptian autistic children, and was associated with other autistic behaviours, including stereotypic behaviours. Moreover, using the German version of the Glasgow sensory questionnaire Jakob et al., [17] found higher affective reactivity in autistic adults as well. Collectively, these findings affirm He et al.,’s taxonomy by demonstrating that affective reactivity is a distinct and impactful dimension of sensory processing in autism, with implications for development, mental health, and intervention. Preliminary research using a case study has documented successful CBT based intervention for noise hypersensitivity in an autistic teenager, underscoring the clinical relevance of affective reactivity [18].

## Perceptual Sensitivity

Perceptual sensitivity refers to individual differences in the detection and discrimination of sensory stimuli at the perceptual level using laboratory based direct assessment measures. Unlike questionnaire-based measures of sensory reactivity, psychophysical approaches measure basic perceptual ability as distinct from the self-reported or caregiver-reported affective and behavioural responses. While psychophysical tasks are certainly associated with specific neural mechanisms, unlike neuroimaging measures that index cortical responses and structural and functional brain connectivity, perceptual sensitivity is typically assessed through psychophysical paradigms in which the stimulus space is carefully mapped. While this is an artificial distinction, it is a useful construct for exploring the levels of sensory reactivity to then inform a holistic understanding.

The extant sensory perceptual literature demonstrates a complex pattern due to the inherent differences from using unique paradigms in different labs to assess a similar ability, the inherent perceptual differences in abilities in autistic and non-autistic individuals, and the inherent differences in processing sensory information across different domains (e.g. auditory or visual) within a single individual. However, the literature suggests that at the perceptual level, autistic sensory perception is characterised not by global hyper- or hyposensitivity, but by domain-specific alterations with both affected and enhanced discrimination dependent on modalities, impaired adaptation mechanisms, and altered temporal processing that can be measured and can inform understanding of groups as well as individuals. While it remains difficult to disentangle the impact of attention, these measures importantly allow researchers to study whether autistic people experience the world differently even without associated valence.

In the tactile domain, detection threshold findings are mixed and depend heavily on the exteroceptive stimulus parameters. Recent work has suggested perceptual differences should be viewed on a more nuanced level, with distinct tasks showing differences. In particular studies have found higher flutter (low-frequency) thresholds, or reduced ability to detect and recognize the change in pattern for slower repeated stimuli applied to the fingers, as well as less adaptation as the stimuli continues over a time course of milliseconds to seconds ([19]; J. L. He et al., [20], Tavassoli, [21] consistent with prior work. Differences in social touch perception have also been observed [22, 23]. Developmental differences over the lifespan are also critical to explore but generally understudied in a longitudinal design. Based on the above, while autistic adults are more likely to show similar tactile thresholds or even enhanced abilities, autistic children often show less acute tactile perception and less habituation over time.

Understanding tactile perception has informed our understanding of affective experience and behavioural responses. Recent studies have linked higher tactile perceptual thresholds to increased sensory reactivity, suggesting that perceptual differences contribute to higher order sensory reactivity as measured with questionnaires (J. L. He et al., [20]; McKernan et al., [24].; Powell et al., [12]. Furthermore, we are learning the neurochemical and neural mechanisms associated with these perceptual observations. Neurochemically, reduced GABA and increased glutamate levels correlate with tactile perceptual differences in both children, and adults (J. He et al., [25]; L.-A. Sapey-Triomphe et al., [26]. However, the relationship is not straightforward; It might be expected that higher GABA is associated with more suppression of sensory input. These studies might reflect an autism-related developmental differences in how excitation and inhibition relate to sensory processing.

Visual perception studies continue to reveal duration-dependent effects, with recent work refining earlier accounts. Manning and colleagues used drift diffusion perceptual modelling combined with EEG to show that autistic children demonstrate typical or even enhanced integration of motion information; differences in motion coherence performance appear to reflect reduced noise exclusion rather than impaired signal integration [27, 28]. A meta-analysis confirmed that global motion processing differences, while reliable, show only small effect sizes with substantial individual variability [29]. Contrast sensitivity work suggests different developmental trajectories, with autistic children showing continued development at high spatial frequencies beyond typical ages [30]. While findings remain heterogeneous, they increasingly suggest that on average there is a challenge with filtering relevant signals from noise, further impacted by the speed of information presentation.

Less work continues in the auditory domain in recent years, though recent meta-analytic evidence supports enhanced pitch perception in autism, particularly with simple tones and in those with language delay history [31]. Dumont and colleagues found that enhanced pitch discrimination was most pronounced in autistic children who had unexpectedly acquired a second language through screen exposure, suggesting that excessive perceptual precision may facilitate alternative language-learning pathways in some individuals [32]. Temporal processing remains affected in a modality-specific manner: Chan and colleagues [33] replicated elevated auditory temporal-order thresholds using online assessment, with autistic children requiring substantially longer intervals to accurately judge temporal order.

Cross-modal temporal processing differences are now understood to be developmentally dynamic. While meta-analytic work confirms wider temporal binding windows in autism [34], longitudinal and cross-sectional studies demonstrate that these differences narrow with age. Ainsworth and Bertone [35]found that binding windows were negatively correlated with age only in autistic participants, with group differences diminishing by adolescence, consistent with evidence by Jertberg and colleagues that McGurk susceptibility is similar to non-autistic adults, with age increasing susceptibility in both groups [36]. Zhou and colleagues found that reduced visual temporal acuity correlated with sensory avoidance specifically in adolescents with higher autistic traits, linking temporal processing to broader sensory experiences [37].

Finally, it is worth noting that questionnaire-based measures of sensory sensitivity, such as the Sensory Perception Quotient, capture subjective perceptual experiences rather than objective thresholds. Taylor and colleagues used a revised SPQ scoring to demonstrate greater hypersensitivity in autistic females, supporting enhanced perceptual functioning accounts [38]. However, psychophysical thresholds and direct assessment measures of sensory behaviour do not consistently correlate with questionnaire responses, suggesting these approaches may index different constructs one measuring perceptual resolution, or even within-individual differences, the other capturing the subjective salience of sensory experiences [39, 40]. Both perspectives are valuable but should not be conflated.

In summary, whilst the psychophysical literature is heterogeneous, it provides evidence that autistic individuals do experience the world differently at a fundamental perceptual level, not merely responding differently to equivalent percepts. However, these differences are highly domain- and task-specific, with enhanced sensitivity in some paradigms, reduced sensitivity in others, and equivalent performance in many. Thus, factors of critical importance are regarding stimulus parameters and design, modality of preference, stimulus domain, but also, the developmental impact. This pattern suggests that simple accounts of global hyper- or hyposensitivity are insufficient, and that higher-order factors, including attention, prediction, and contextual modulation, shape how basic perceptual differences manifest across tasks across development. A critical gap remains in understanding how and under what circumstances these perceptual differences relate to the sensory reactivity captured by questionnaire measures and reported in daily life. The field would benefit from studies that directly link psychophysical thresholds to affective and behavioural responses within the same individuals and across the lifespan. Additionally, this literature is largely limited to older children, adolescents, and adults without intellectual disability, given the demands of traditional psychophysical paradigms. Encouragingly, approaches are being developed to extend perceptual assessment to younger children, minimally verbal individuals, and those with co-occurring intellectual disability, which will be essential for understanding sensory processing across the full spectrum.

## Physiological Responsivity

Green and colleagues [6] identified a subgroup of autistic children with elevated parent-reported and observed Sensory Over Responsivity (SOR) who also exhibited heightened sympathetic arousal, suggesting that behavioural responsivity may be underpinned by autonomic dysregulation. Complementary findings by Matsushima et al., [41]demonstrated increased vagal activity in response to less controllable sensory stimuli, indicating that behavioural rigidity may be linked to atypical physiological modulation.

Technological innovations, such as the virtual reality system developed by López-Carral et al., [42], provide ecologically valid platforms for simulating sensory environments and capturing real-time behavioural responses. These systems offer promising avenues for disentangling the dynamic interplay between sensory input and behavioural output. Furthermore, motoric expressions of behavioural responsivity have been observed in studies of postural control and gait. Cham et al., and Bick et al., [43, 44] reported that autistic individuals exhibit increased postural sway and reduced gait adaptability under sensory and attentional load, reflecting diminished sensorimotor integration and behavioural flexibility.

Pharmacological interventions targeting neural excitability, such as bumetanide, have shown preliminary efficacy in reducing irritability associated with sensory processing difficulties [45], suggesting potential modulation of behavioural responsivity through GABAergic mechanisms. Furthermore, preliminary data upregulating the parasympathetic nervous system through cranial electrotherapy stimulation has demonstrated reduced sensory sensitivity, anxiety and emotional regulation [46]. Collectively, these findings underscore the importance of distinguishing behavioural responsivity as a discrete construct within sensory processing frameworks and highlight the need for multimodal methodologies to elucidate its underlying mechanisms and inform targeted interventions.

## Structural and Functional Neural Mechanisms of Sensory Reactivity

Neural mechanisms of autism in general, and sensory reactivity in particular, have been explored for decades [47]. Extraordinary variability in structural and functional data for autistic individuals, while not unexpected, has hampered progress such that important neural differences, related to the underlying etiology rather than the autism symptom cluster, get obscured by averaging elevated and reduced brain volumes, white matter connectivity, and network coherence. Furthermore, there is a growing recognition that the intrasubject neuroimaging variability or “noise” [48] may be part of the explanation for the functional differences [49]. This is a reminder that for neural transmission of sensory information, the timing of firing matters; speed, consistency, as well as the oscillatory frequency contribute to the modulation of the incoming signals [50]. Thus, neuroanatomic studies that explore sensory reactivity as a coalescing phenotype, rather than autism more broadly, have become more common and led to additional insights.

Structural investigations of autism and sensory differences include studies of genetic and toxic exposure models, postmortem human pathology, and in vivo neuroimaging. Fundamentally, any disruption of neuronal organization [51, 52], neuronal projections [53, 54], and/or electrochemical transmission [26, 55–58], whether it be due to a genetic alteration (e.g. Fragile X [59], brain injury from prematurity [60] or toxic exposures can alter the flow of sensory information coming from inside (interoception) or outside (exteroception) of the body and lead to misregistration and misinterpretation of the signal [61, 62]. This is particularly true if the neural disruption involves critical networks for sensory processing. Not surprisingly, emerging data across models of sensory processing point to the brainstem, cerebellum [63], subcortical structures [64, 65], sensory cortices (primary and multisensory neuronal hubs in association cortices [66] as core links in the sensory chain. Diffusion Tensor Imaging which measures the integrity of white matter connections between the cortical brain areas has now repeatedly shown reduced organization in the posterior and interhemispheric (corpus collosum) tracts. This structural difference has been correlated with sensory reactivity and emotional regulation joining the neural mechanism to observable behaviour. While the past decade has highlighted the importance of moving from measuring discrete brain areas to brain networks [67–69], the next decade will contribute more granular information emerging from organoid and animal models, machine learning algorithms, and ultrahigh field (7 tesla) brain mapping.

Functional investigations of brain activity have seen an exponential increase with the advent of functional connectivity Magnetic resonance Imaging (fcMRI), magnetoencephalography (MEG), transcranial magnetic stimulation, and wearable devices for round the clock assessment. Brain network activity continues to echo a core hypothesis, espoused in 2003 and updated in 2019 by John Rubenstein and Michael Merzenich, that sensory based traits result from an imbalance in excitation and inhibition [70] (the E: I balance) at neuron and circuit levels. At a cortical network level, this imbalance has been recognized as early as 6 weeks of age in infants at risk for autism, persists across the lifespan, and has been associated with restricted and repetitive behaviour and sleep differences [71–73]. The E: I balance has been a guiding principle in autism and is equally important in understanding the mechanisms of sensory reactivity.

In concert with structural findings for sensory reactivity, functional activity often implicates reciprocal network connections between the brainstem, cerebellum, subcortical and sensorimotor cortical regions in mediating sensory reactivity. EEG findings support differences in sensory processes in autistic children and adults, with differences found in sensory gating, adaptation, and in response to GABAergic intervention across developmental stages [71, 74–76]. In a recent resting state fcMRI study, children with directly measured sensory over responsivity exhibit reduced long-range functional connectivity across all brain networks but the short-range pattern is more complicated, but also more informative [77]. Sensory reactive children show decreased “local” functional connectivity in exogenous networks (networks receiving sensory information originating from outside the brain), but increased functional connectivity in endogenous networks (networks processing information within the brain). By contrast, children without sensory reactivity show a mirrored, or exactly opposite, pattern–increased exogenous and decreased endogenous connectivity. Bridging from neural activity to real world behaviour, there is a more nuanced splitting of this observed pattern. The sensory reactive and emotionally resilient children demonstrate a balanced functional connectivity, with equal and opposite “turning down” the exogenous and “turning up” the endogenous functional connectivity. However, the sensory reactive but emotionally dysregulated children show no difference in exogenous and endogenous network functional connectivity. This finding suggests that a dual brain network approach may help understand and potentially guide behavioural treatment for sensory reactive individuals and having a balanced contrast, not equal activity, in the exogeneous and endogenous systems is critical for resilience. Thus, a treatment approach to change brain network activity with developmental therapies, neuromodulation [78], or neurochemistry must be tailored by whether the individual is sensory reactive or not.

## Conclusion

Sensory reactivity in autism is a multidimensional phenomenon that spans behavioural, affective, perceptual, physiological, and neural levels. Recent advances underscore that these dimensions are interrelated yet distinct, each contributing unique insights into how autistic individuals experience and respond to their environments. Behavioural and affective responsivity shape daily functioning and mental health, while perceptual differences reveal fundamental alterations in sensory encoding that cannot be explained by global hyper- or hyposensitivity alone. Physiological and neural findings highlight the role of autonomic regulation and excitation–inhibition balance, offering mechanistic pathways. Despite significant progress, critical gaps remain: the need for longitudinal, multimodal studies that integrate these levels within individuals, across development, and in diverse populations. Future research should prioritize ecological validity, harmonize measurement approaches, and explore interventions that target both environmental supports and neurobiological mechanisms. By embracing this integrative framework, the field can move toward a more precise understanding of sensory reactivity and its cascading effects, ultimately informing personalized strategies to enhance quality of life for autistic individuals.

##  Key References

- He, J. L., Williams, Z. J., Harris, A., Powell, H., Schaaf, R., Tavassoli, T., & Puts, N. A. J. (2023). A working taxonomy for describing the sensory differences of autism. *Molecular* *Autism*, *14*(1), 15. 10.1186/S13229-022-00534-1.

◌ He et al. (2023) highlight how interchangeable terminology has hindered the scientific understanding of sensory differences in autism. They propose a hierarchical taxonomy to clarify distinctions among terms such as sensory sensitivity, affective reactivity, and behavioural responsivity, aiming to improve conceptual precision and guide future research.

- Verhulst et al. (2022). The Perceived Causal Relations Between Sensory Reactivity Differences and Anxiety Symptoms in Autistic Adults. *Autism in Adulthood*, *4*(3), 183–192. 10.1089/AUT.2022.0018.

◌ Verhulst et al., (2022) asked autistic adults what they think comes first sensory reactivity differences or anxiety. They showed that autistic adults perceive sensory hyperreactivity to precede anxiety and sensory seeking to be more a cause of it.

## Data Availability

No datasets were generated or analysed during the current study.
